# Adoption of left bundle branch area pacing using stylet-driven lead in a tertiary academic training center: Learning curve and acute procedural outcomes

**DOI:** 10.1016/j.hroo.2024.12.009

**Published:** 2024-12-27

**Authors:** Suraya Hani Kamsani, John L. Fitzgerald, Anand Thiyagarajah, Shaun Evans, Mohanaraj Jayakumar, Jonathan P. Ariyaratnam, Varun Malik, Catherine O’Shea, Bradley M. Pitman, Christopher X. Wong, Mehrdad Emami, Glenn D. Young, Dennis H. Lau

**Affiliations:** 1 Department of Cardiology, Royal Adelaide Hospital, Adelaide, South Australia, Australia; 2 Department of Cardiology, National Heart Institute, Kuala Lumpur, Malaysia; 3 Australian Dysautonomia and Arrhythmia Research Collaborative, The University of Adelaide, Adelaide, South Australia, Australia; 4 Faculty of Health and Medical Sciences, The University of Adelaide, Adelaide, South Australia, Australia

**Keywords:** Left bundle branch area pacing, Conduction system pacing, Stylet-driven leads, Cardiac pacing, Learning curve

## Abstract

**Background:**

Left bundle branch area pacing (LBBAP) has recently emerged as a strategy for conduction system pacing.

**Objective:**

The purpose of this study was to evaluate the initial learning experience and acute procedural success in adopting this procedure in an academic training center.

**Methods:**

A retrospective review of LBBAP procedures using the Biotronik Solia stylet-driven lead from June 2022 to December 2023 was performed. Procedural and fluoroscopy times with electrocardiographic and pacing parameters were evaluated to determine safety and acute procedural outcomes.

**Results:**

A total of 69 patients (mean age 75 ± 12 years; 60.9% male) underwent LBBAP implantation over 18 months for standard pacing indications by 10 implanters (including 7 fellows-in-training) without previous experience in LBBAP technique. Mean total procedural time was 74.1 ± 23.5 minutes, and mean fluoroscopy time for LBBAP lead insertion was 9.3 ± 5.4 minutes. Mean paced QRS duration was 115.2 ± 15.5 ms, and mean left ventricular activation time was 79.4 ± 14.5 ms. An rsRʹ pattern was achieved in 76.8%. LBBAP was successful in 78.3% (overall 43.5% single deployment; median 2 [interquartile range 1–3]) with excellent LBBAP lead parameters: threshold 0.8 ± 0.4 V at 0.4 ms; sensing 9.4 ± 4.2 mV; impedance 627 ± 131 Ω. Acute procedural complications included damaged lead helix requiring a second lead (4.3%), pneumothorax (2.9%), and acute LBBAP lead dislodgment (1.4%). Septal perforation occurred in 10.1% of cases with no acute sequelae. When analyzed in tertiles, the number of lead deployment attempts was significantly reduced with no changes to procedural success rates with increasing experience.

**Conclusion:**

Adoption of LBBAP with stylet-driven lead in an academic training center is feasible and safe, with satisfactory success rates and no overly steep learning curve.

**Key Findings**

▪ Real-world adoption of left bundle branch area pacing procedure in a low- to medium-volume tertiary center is feasible with acceptable success rates.
▪ With increasing operator experience, there was a reduction in the number of lead deployment attempts as well as a trend toward shorter procedural time.
▪ Overall acute procedural complication rates were low.

## Introduction

Over the past decade, there has been a surge in the adoption of cardiac physiological pacing, specifically with His-bundle pacing (HBP) and left bundle branch area pacing (LBBAP). This shift has been prompted by the identification of adverse long-term effects associated with traditional right ventricular pacing, including the development of pacing-induced cardiomyopathy, atrial fibrillation, and a subsequent increase in mortality rates.^1^, ^2^, ^3^ Although HBP procedures have been instrumental in addressing these concerns, they come with their own set of challenges, marked by a long learning curve and notable occurrences of rising capture thresholds over time.^4^^,^^5^ In contrast, LBBAP has emerged as a promising alternative, demonstrating a higher rate of procedural success coupled with consistently low capture thresholds and potentially more stable lead parameters.^6^^,^^7^

Several previous observational studies have affirmed the feasibility and safety of LBBAP, establishing it as a viable option with clinical outcomes comparable to those achieved through HBP.^7^, ^8^, ^9^, ^10^, ^11^, ^12^ More recently, in a large cohort study of 1778 patients with heart failure with reduced ejection fraction (left ventricular ejection fraction [LVEF] <35%), LBBAP was found to be advantageous over traditional biventricular pacing in significantly narrowing the QRS duration, resulting in a greater improvement in LVEF and reduction in primary composite outcomes of death and heart failure hospitalizations.^13^ Similar to HBP implantation, lumenless leads (LLLs) were the initial lead of choice for LBBAP implantation.^7^^,^^8^ Recently, LBBAP using standard stylet-driven leads (SDLs) also has been demonstrated to be feasible and safe with advantages over LLL due to enhanced stylet support, providing better torquability and facilitating easier septal penetration.^12^^,^^14^^,^^15^

The aim of this study was to determine the feasibility, safety, and learning curve in new LBBAP implanters using SDL within the initial 18 months of adoption into clinical practice at a tertiary teaching hospital. The evaluation encompasses various key parameters, including procedural time, fluoroscopy time, electrical parameters during implant, percentage of LBBAP success, and any periprocedural complications.

## Methods

### Study patients

In this retrospective observational study, all consecutive patients with established pacing indications who underwent LBBAP procedures with SDL by 10 implanters between June 2022 and December 2023 at our tertiary academic training hospital were included. This study was approved by the local Human Research Ethics Committee and adhered to the ethical standards and principles for medical research in the Declaration of Helsinki. Due to the retrospective nature of this work with de-identified data, patient consent was not required. Baseline characteristics of the patients, including medical history, pacing indications, and transthoracic echocardiographic and electrocardiographic parameters, were obtained from the electronic medical records.

### LBBAP implantation procedure

All procedures were performed by 10 implanters (including 7 fellows-in-training) without previous experience in LBBAP technique using the 5.6F SDL with extendable helix (Solia S60, Biotronik SE & Co., Berlin, Germany). The new implanters were supervised by an attending physician who has completed a clinical observership in another high-volume center and supported by a trained device specialist. The delivery of this lead was facilitated through a preshaped sheath (Selectra 3D, Biotronik SE & Co.). The choice of sheath curve (55 or 65) and length (39 or 42 cm) was based on the cardiac anatomy and venous access site. Before implantation, the lead was first prepared by extending the helix in advance and subsequently maintaining the tension by keeping the stylet guide connected and turned 8–10 times clockwise. This additional preparation is necessary to prevent helix retraction during lead deployment. The lead is subsequently advanced inside the sheath across the tricuspid valve with fluoroscopic guidance in the left anterior oblique 20°–30° projection, targeting the high and middle septal areas. Unipolar pace mapping was performed in these areas, observing for the development of “W” pattern on lead V_1_ of surface electrocardiogram. Once this was identified, the lead helix was advanced into the septum with initial rapid rotation. The depth of septal penetration was confirmed with contrast injection via the Selectra 3D sheath. Electrical parameters including R-wave amplitude, unipolar pacing capture threshold, and impedance as well as left ventricular activation times (LVATs), morphology, and duration were checked frequently to guide lead deployment. Further rotation at a slower pace was then performed to ensure the entire lead helix was properly embedded in the septum. Throughout this process, the current of injury and lead impedance were carefully observed to avoid unwanted ventricular septal perforation. The lead was repositioned if pacing parameters were unsatisfactory or in the presence of possible septal perforation with negative injury current and drop in lead impedance. LBBAP procedural success was defined as the presence of any 2 of the following criteria: (1) paced QRS complex (≤130 ms); (2) presence of rsRʹ pattern of the paced QRS complex in lead V_1_; and (3) LVAT ≤80 ms.^16^, ^17^, ^18^

### Data collection

Details of the LBBAP procedure, including deployment attempts, fluoroscopy times for LBBAP lead deployment, and total procedural times were based on procedural reports. Capture threshold, R-wave amplitude, and impedance were measured during implantation. Other electrical parameters defining LBBAP success also were collected. Acute procedural complications including lead damage and dislodgment, septal perforation, and pneumothorax were documented.

### Statistical analysis

Descriptive statistics for continuous variables are given as mean ± SD or median [interquartile range] according to their distribution. Categorical variables are summarized with number (percentage). To evaluate the learning experience across different implanters, the study cohort was divided into 3 tertiles—group 1 (n = 24): the first 3 procedures from each implanter; group 2 (n = 22): the 4th to 9th procedures; and group 3 (n = 23): the 10th procedure onward. The differences between these groups were assessed using 1-way analysis of variance or Kruskal-Wallis test for continuous variables and χ^2^ or Fisher exact test for categorical variables. *P* <.05 was considered significant. All statistical analysis was conducted with IBM SPSS Statistics Version 28.0 (IBM Corp., Armonk, NY).

## Results

### Baseline characteristics

During the 18-month study period, LBBAP procedures using the prespecified SDL were attempted in a total of 69 patients. Use of LBBAP was at operator discretion and accounted for 12.4% (69/555) of all pacemaker implants at our institution during this period.

Detailed baseline patient characteristics are outlined in Table 1. Mean age of our study cohort was 75 ± 12 years, and 46 of the patients (60.9%) were male. Mean LVEF was 57% ± 10%, with 17.4% of patients having LVEF ≤50% before device implant. Pacing indications were sinus node disease (47.8%), atrioventricular (AV) block (30.4%), conduction disorders without AV block (13.0%), and pre-AV node ablation in patients with atrial fibrillation (8.7%). Seventeen patients (24.6%) were in atrial fibrillation at the time of the implant procedure. Right bundle branch block was identified at baseline in 26.1%, intraventricular conduction delay in 10.1%, and left bundle branch block in 7.2%. Mean baseline QRS duration was 121 ± 29 ms.

**Table 1:** Baseline characteristics of the study cohort (N = 69)

	
Age, y	75.2 ± 11.5
Female	27 (39.1)
Comorbidities	
Hypertension	46 (66.7)
Atrial fibrillation/flutter	31 (44.9)
Coronary artery disease	30 (43.5)
Diabetes mellitus	22 (31.9)
Heart failure	18 (26.1)
Previous stroke/TIA	6 (8.7)
Pacing indication	
Sinus node disease	33 (47.8)
AV block	21 (30.4)
Conduction disorders without AV block	9 (13.0)
Pre-AVN ablation	6 (8.7)
Echocardiography	
Baseline LVEF, %	57.4 ± 10.4
LVEF <50%	12 (17.4)
Moderate–severe TR, n (%)	12 (17.4)
Baseline rhythm	
Sinus rhythm/bradycardia	34 (49.3)
Atrial fibrillation	17 (24.6)
Junctional rhythm	1 (1.4)
Paced rhythm	1 (1.4)
ECG parameters	
QRS duration, ms	121.1 ± 28.5
QRS morphology	
Normal	38 (55.1)
RBBB	18 (26.1)
IVCD	7 (10.1)
LBBB	5 (7.2)

Values are given as mean ± SD or n (%).

AV = atrioventricular; AVN = atrioventricular node; ECG = electrocardiography; IVCD = intraventricular conduction delay; LBBB = left bundle branch block; LVEF = left ventricular ejection fraction; RBBB = right bundle branch block; TIA = transient ischemic attack; TR = tricuspid regurgitation.

### LBBAP procedural characteristics

LBBAP procedural success was achieved in 78.3% (n = 54) of the cohort (Table 2). LBBAP procedure was abandoned in 2 cases because of inability to reach the desired septal region with 2 different sheaths in 1 patient and LBBAP lead dislodgment after atrial lead insertion in the other patient. Mean total procedural time was 74.1 ± 23.5 minutes, and mean fluoroscopy time for LBBAP lead insertion was 9.3 ± 5.4 minutes. Overall, 43.5% of cases required only a single deployment attempt, with an overall median 2 [1–3] attempts.

**Table 2:** Procedural characteristics and outcome (N = 69)

	
Procedural characteristics	
Procedural time, min	74.1 ± 23.5
Fluoroscopy time, min	9.3 ± 5.4
Lead deployment attempt, n	2 [1–3]
Acute LBBAP success	54 (78.3)
Acute complication	
Septal perforation	7 (10.1)
Lead damage	3 (4.3)
Pneumothorax	2 (2.9)
Lead dislodgment	1 (1.4)
Electrophysiological parameters	
Baseline QRS duration, ms	121.1 ± 28.5
Paced QRS duration, ms	115.2 ± 15.5
ΔQRS duration, ms	–6.0 ± 24.2
Stim-LVAT, ms	79.4 ± 14.5
V_6_–V_1_ interpeak interval, ms	48.4 ± 20.5
Right bundle branch pattern	53 (76.8)
Pacing parameters	
Ventricular lead capture threshold, V	0.8 ± 0.4
Sensed R-wave amplitude, mV	9.4 ± 4.2
Ventricular pacing impedance, Ω	627.0 ± 130.8

Values are given as mean ± SD, median [interquartile range], or n (%).

LBBAP = left bundle branch area pacing; ΔQRS duration = change in QRS duration post LBBAP; Stim-LVAT = stimulus to left ventricular activation time.

Mean paced QRS duration was 115 ± 16 ms with rsRʹ pattern demonstrated in 76.8% (Table 2). Mean LVAT was 79 ± 15 ms, and mean V_6_–V_1_ interpeak interval was 48.4 ± 20.5 ms. LVAT <80 ms was achieved in 56.7% (n = 38), and V_6_–V_1_ interpeak interval >40 ms was achieved in 59.7% (n = 40). LBBAP lead parameters were satisfactory and as follows: threshold 0.8 ± 0.4 V at 0.4 ms; sensing 9.4 ± 4.2 mV; impedance 627.0 ± 130. 8Ω (Table 2). Overall, paced QRS duration exhibited a significant reduction compared to the baseline values (pre 121 ± 29 vs post 115 ± 16 ms; *P* <.05). In comparison to the baseline measurements, 54.4% of patients showed improvement with a mean difference of –24 ± 17 ms, 44.1% experienced widening of QRS with an increase of +16 ± 10 ms, and 1.5% (n = 1) exhibited unchanged QRS duration of 124 ms.

### Learning experience of LBBAP procedures

**Figure 1:**
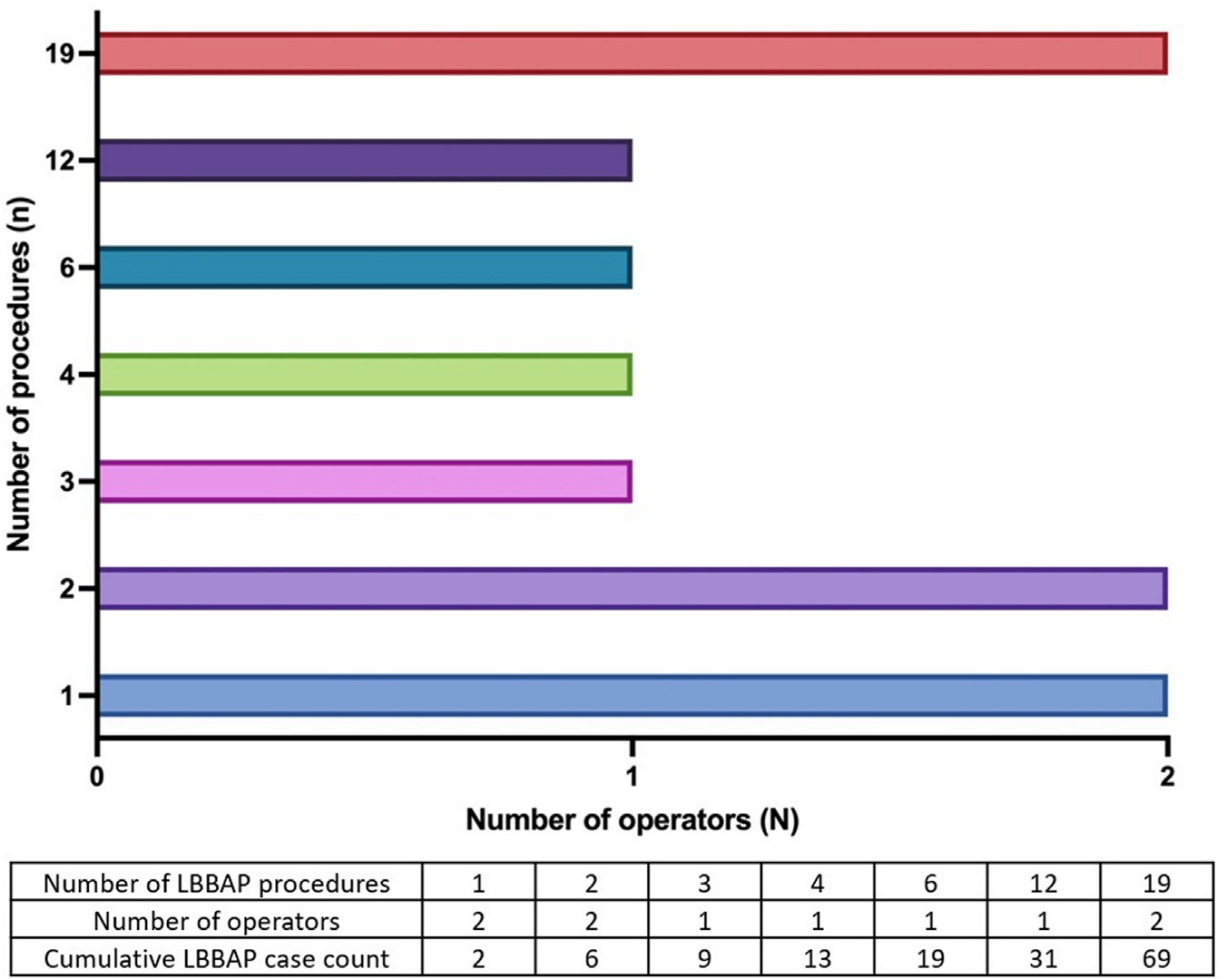
Number of left bundle branch area pacing (LBBAP) implantation procedures per operator. Spread of number of LBBAP implants performed by the 10 operators in this series is shown. Five operators performed ≤3 procedures each, and 5 operators performed ≥4 procedures, with 3 of these operators having performed ≥12 procedures.

**Figure 2:**
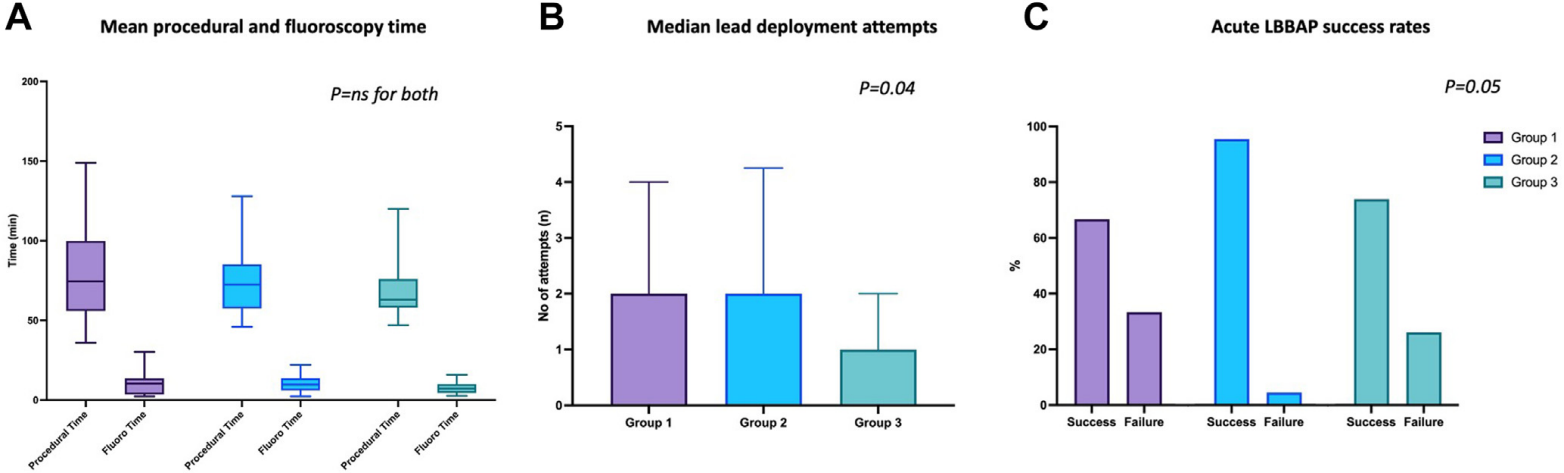
Procedural characteristics according to experience. With increasing left bundle branch area pacing (LBBAP) implant experience, there was no significant differences in procedural and fluoroscopy times **(A),** reduced number of lead deployments **(B),** and trend to higher LBBAP success rate **(C).**

Median number of procedures per implanter was 3.5 [2–12], with 4 implanters having performed at least 6 LBBAP implants (Figure 1). Procedural time was shorter as operators gained more experience, although this did not reach statistical significance (group 1: 78.7 ± 30.2 minutes vs group 2: 75.9 ± 22.0 minutes vs group 3: 68.3 ± 16.0 minutes; *P* = .32) (Figure 2). Fluoroscopy time for LBBAP lead implantation did not differ with increasing experience (group 1: 10.0 ± 6.9 minutes vs group 2: 10.0 ± 5.2 minutes vs group 3: 7.9 ± 3.8 minutes; *P* = .40). However, lead deployment attempts were significantly lower with increasing experience (group 3: median 1 [1–2] vs group 2: 2 [1–4.25] vs group 1: 2 [1–4]; *P* = .04). Acute procedural success rate was not significantly different between the groups (group 1: 66.7% vs group 2: 95.5% vs group 3: 73.9%; *P* = .05). Overall acute complication rates also did not differ between the groups, but septal perforation (n = 7) occurred more frequently in the less experienced groups (group 1: n = 2; group 2: n = 4) and reduced to 1 in group 3.

### Procedural complications

Acute procedural complications included septal perforation (n = 7 [10.1%]), damaged lead helix requiring a second lead (n = 3 [4.3%]; none abandoned or left *in situ*), pneumothorax (n = 2 [2.9%]), and acute LBBAP lead dislodgment (n = 1 [1.4%]). All cases of septal perforation were identified with negative current of injury during lead deployment and without any sequelae after repositioning. No pericardial effusion was encountered in the entire cohort on postimplant echocardiogram.

## Discussion

This study highlighted the early experience of LBBAP procedures with SDL by multiple new implanters in a single tertiary center. The key observations from this study are as follows. (1) Adoption of LBBAP procedure in a low- to medium-volume tertiary center is feasible, demonstrating acceptable success rates (78.3%) among multiple new implanters with low rates of acute procedural complications. (2) After the LBBAP procedure, there was a notable and statistically significant reduction in QRS duration. (3) There was a significant reduction in number of lead deployment attempts as well as a trend suggesting improvements in procedural time with increasing operator experience. Procedural duration and QRS improvement in our study were on par with those reported in other studies.^7^^,^^19^^,^^20^ Of note, although our success rates were slightly lower than previously reported, they remain encouraging because previous studies either excluded their early experiences from analysis or solely evaluated the initial experience of a small number (<3) of high-volume operators in a single center.^7^^,^^19^, ^20^, ^21^, ^22^

### Learning curve for LBBAP implants

The first successful case of LBBAP was reported by Huang et al^23^ in a patient with dilated cardiomyopathy who failed both left ventricular lead placement and HBP attempts. They successfully corrected her left bundle branch block with low and stable pacing output, which after 1 year translated into an overall improvement of clinical outcomes. Subsequent multiple observational studies have similarly demonstrated the feasibility and safety of LBBAP as an attractive alternative pacing modality.^6^, ^7^, ^8^, ^9^, ^10^, ^11^^,^^24^^,^^25^ During the initial adoption of HBP, its learning curve was reported to vary between 30 to up to 100 cases, with improvement in procedural duration without affecting the success rates.^4^^,^^26^^,^^27^ Earlier observational studies with LBBAP procedures indicated that the most substantial learning curve occurred within the initial 50–100 cases, with shorter duration of procedure and higher success rates before reaching a plateau. However, these studies , predominantly focused on single operators in high-volume centers and used LLLs in their procedures.^19^^,^^28^

Two recently published studies using the SDL indicated that a plateau phase was attained earlier after 25–50 cases.^20^^,^^29^ Taking these into consideration along with the fact that none of our implanters have performed >20 procedures, it is highly likely that our cohort of new implanters still is within the learning curve, and hence overall LBBAP success is likely to be higher with further increase in operator experience. Nevertheless, our data represent a real-world learning curve in a tertiary academic teaching center, and the adoption of LBBAP technique using SDL seems highly feasible and safe without undue prolongation of procedural time. Notably, septal perforation occurred largely in operators who have performed <10 LBBAP procedures. Taken together, LBBAP using SDL seems to have a shallower learning curve than LLL, perhaps because of increased stiffness with the presence of the stylet to improve torquability and septal penetration, although further dedicated comparative studies are needed.

### Definition of LBBAP

Varying definitions of successful LBBAP have been used in different studies to date. The most recent society guidelines pertaining to cardiac physiological pacing have established a categorization framework for LBBAP based on specific pacing locations and output responses. This classification system encompasses 3 distinct types: selective left bundle branch pacing, nonselective LBBAP, and deep septal pacing.^30^ Previous studies have also provided various practical methods, including specific electrocardiographic and intracardiac electrogram-based criteria to determine left bundle branch capture.^31^, ^32^, ^33^ However, in our study, given the constraints of a relatively small sample size, we opted not to distinguish between the different LBBAP responses but rather focus on the new implant technique and its learning curve. Furthermore, there are insufficient data on long-term differences in the outcomes of specific LBBAP locations.^24^

### Clinical implications

The issue of generalizability of LBBAP to real-world practice has emerged, given that previous studies predominantly focused on a limited number of high-volume centers and highly experienced implanters. In this context, our study serves as a valuable contribution by shedding light on the feasibility and practicality of implementing LBBAP, particularly among new implanters, in a low- to medium-volume training center. It is likely that with future generations of purposeful designed leads and sheaths for LBBAP, the learning curve will become shallower with lower risk of complications such as septal perforation and lead damage. Indeed, longer-term data on current-generation technology regarding durability and performance of SDL in LBBAP remain awaited to inform future practice. Future studies will also help to delineate the relative importance of LBBAP lead positioning and confirmation of conduction system capture on long-term risk of pacing-induced cardiomyopathy.

### Study limitations

Our study is limited by its retrospective, nonrandomized design and small sample size (both number of patients and new implanters). Consequently, the study may lack sufficient power to identify statistically significant differences between the groups. To mitigate potential selection bias, we ensured the inclusion of all consecutive patients during the study period. It is probable that we have not yet reached the plateau phase in our learning curve, given that the maximum number of implants per operator was only 19. We did not include a comparison group with either LLL for LBBAP or control group with traditional right ventricular pacing to evaluate the additional effort required with LBBAP using SDL and to compare procedural duration or fluoroscopy utilization. Additionally, the study exclusively focused on acute procedural outcomes without an extended follow-up period. Although we did not focus on the specificity of conduction system capture in our cases, it is important to highlight that these data reflect the real-world adoption of LBBAP in a single tertiary center with multiple trainee operators.

## Conclusion

Our early experience in an academic teaching center demonstrated that adoption of LBBAP with SDL is feasible and safe, with satisfactory acute success rates. As experience increased, there was a significant reduction in the number of deployment attempts with similar success rates.
